# Estimation and Co-Relation of the Neutrophil Count and Neutrophil Chemotaxis in Patients With Gingivitis, Chronic Periodontitis, and Localized Aggressive Periodontitis Compared With Healthy Controls

**DOI:** 10.7759/cureus.36627

**Published:** 2023-03-24

**Authors:** Megha Vanasi, Shamila Shetty, Vinit B Patel, Rinku Jagnade Saini, Nidha Begum, Ranjita Singh Baghel

**Affiliations:** 1 Department of Periodontics, Faculty of Dental Science, Dharmsinh Desai University, Nadiad, IND; 2 Department of Periodontics, A.J. Institute of Dental Sciences, Mangalore, IND; 3 Department of Periodontics, Index Institute of Dental Sciences, Indore, IND; 4 Department of Periodontics, KVG (Kurunji Venkatramana Gowda) Dental College & Hospital, Sullia, IND

**Keywords:** gingivitis, periodontitis, neutrophil chemotaxis., neutrophil count, localized aggressive periodontitis, chronic periodontitis

## Abstract

Introduction: Neutrophils are the most plentiful WBCs found in human blood. They are the first cells to respond to wounds and foreign invaders in the human body. They help the body fight infections. The neutrophil count may be used to check for infections, inflammation, or any other underlying conditions. The lower the neutrophil count, the higher the infection risk. Chemotaxis is the ability of the body cells to move in a specific direction as a response to a chemical stimulus. Neutrophil chemotaxis, a feature of the innate immune response, is the directed migration of neutrophils from one site in the body to another to provide effector functions. The present study was aimed at estimating and co-relating the neutrophil count and neutrophil chemotaxis in patients who had gingivitis, chronic periodontitis, and localized aggressive periodontitis, and in healthy participants.

Methods: Eighty participants (40 males and 40 females), aged 20-50 years, were included in the study and divided into four groups: Group I: control group with healthy periodontium; Group II: participants with gingivitis; Group III: participants with periodontitis; and Group IV: participants with localized aggressive periodontitis. Blood samples were collected for hematological analysis to evaluate the neutrophil counts and neutrophil chemotaxis.

Results: The mean value of neutrophil count (%) was highest in Group IV (72.535) followed by Group III (71.29), Group II (62.13), and least in Group I (58.15). This difference is statistically significant (p < 0.001). On intergroup comparison, a statistically significant difference was noted between all the groups except between Group I and Group II, and between Group III and Group IV. The mean value of neutrophil count and neutrophil chemotaxis assay were found to be statistically significant in all four groups.

Conclusion: This study shows a positive correlation between neutrophils and periodontal diseases which could be beneficial for further studies.

## Introduction

Periodontitis is a disease attributable to plaque, bacterial pathogens, altered inflammatory mediators, and immune responses [1]. Periodontitis can deteriorate systemic conditions through the pathology caused by neutrophils, which have a crucial role in the process of inflammation and pathogenesis by the release of cytokines [2]. As reported, aggressive periodontitis is associated with impaired neutrophil count and chemotaxis, which emphasizes the importance of neutrophils in the origination and development (pathogenesis) of periodontal disease [3]. Research has shown that periodontitis is directly correlated to an increased risk of various cardiovascular diseases [4], and the correlation between coronary artery disease and neutrophil count is both an independent risk factor and a prognostic indicator [5].

The current study was performed to estimate and correlate the neutrophil count and chemotaxis in patients with gingivitis, chronic periodontitis, and localized aggressive periodontitis (LAP), and healthy participants. It would also help explain how periodontitis and the emergence of cardiovascular illnesses are related.

## Materials and methods

Eighty participants (40 males and 40 females) were selected from the Department of Periodontics, A.J. Institute of Dental Sciences, Mangalore, Karnataka, India, from 2016 to 2019. Ethical clearance was obtained from A.J. Institute of Dental Sciences (approval number: CR/348/Inst/LA/2013) and informed consent was acquired from every patient. A complete case history of the patients was documented. This was followed by recording various indices and examining radiographs for those patients who showed clinical attachment loss. Based on the aforementioned observations, the participants were split into four groups: Group I: control group consisting of 20 participants with healthy periodontium; Group II: gingivitis group consisting of 20 subjects with moderate to severe gingivitis, according to Loe and Sillness gingival index (GI); Group III: periodontitis group consisting of 20 subjects exhibiting a moderate to severe form of chronic periodontitis (CP); and Group IV: periodontitis group consisting of 20 subjects with LAP.

Complete medical and dental case histories of the participants were recorded. The plaque index (PI) was recorded in accordance with the criteria given by Löe [6]. The GI was recorded in accordance with the criteria given by Löe [6]. The calculus index simplified (CI-S) was recorded according to the criteria given by Green and Vermillion [7]. Clinical parameters such as the determination of probing pocket depth and the level of clinical attachment are determined using William’s graduated periodontal probe and radiographs (Orthopantomogram (OPG)).

Inclusion and exclusion criteria

The inclusion criteria or the four groups were as follows: (a) Group I: participants who were systemically healthy and had an absence of any periodontal disease; (b) Group II: participants with bleeding on probing and diagnosed with moderate to severe gingivitis (gingival index score >1.1); (c) Group III: participants with at least three sites with a probing depth greater than or equal to 5mm and at least three sites with attachment loss > or equal to 5mm. Loss of attachment and amount of tissue destruction should be in accordance with local factors, including plaque and calculus and radiographical visibility of bone loss, horizontal or vertical; (d) Group IV: participants showing accelerated loss of attachment and destruction of bone. Amount of periodontal destruction should be inconsistent with local factors, systemically healthy individuals, and familial aggregation of cases (optional). Onset around the time of puberty with damage to periodontal tissues localized to permanent incisors and first molars and arc-shaped loss of alveolar bone from the distal surface in the second premolar to the mesial surface in the second molar or involving the incisors should be seen radiographically. 

Patients presenting systemic conditions that could change the aforementioned parameters, pregnant or nursing women, and participants who had recently undergone periodontal therapy (in under six months) were all excluded from the study. Patients with the use of any antibiotics or anti-inflammatory drugs within the previous three months were excluded. Patients with other infections that could influence any of the aforementioned study parameters, such as the flu, the common cold, or any other ENT infections, etc., participants with a history of drinking, smoking, and chewing tobacco, patients who had previously experienced neutrophil defects such as leukocyte adhesion deficiencies, hyperimmunoglobulin E syndrome, Chediak-Higashi syndrome, neutrophil-specific granule deficiency, chronic granulomatous disease, and myeloperoxidase deficiency were excluded.

The neutrophil count and the neutrophil chemotaxis assay were analyzed. In an aseptic environment, 6 ml of arterial blood was drawn in a single-use syringe by venipuncture from the antecubital fossa and was relocated to two separate vials, one of which contained an anticoagulant. The samples were then transported to the laboratory. The peripheral samples of blood were obtained from the antecubital fossa by venipuncture at the clinicopathological laboratory of the institution. The samples were analyzed for the following: total leukocyte count (TLC) (cells/cubic mm), in which, the neutrophil count was analyzed using a hematological automated analyzer (Sysmex XN-1000™, Sysmex Corporation, Kobe, Hyogo, Japan) in the institution, which used the principles of volume, conductivity, scatter (VCS), and fluorescence.

Neutrophil chemotaxis assay

Samples of blood collected were stored in vials with anticoagulants. These were combined with equal quantities of minimum essential medium (MEM), which contains Hank’s balanced salt solution and 6% dextran, about 3-5 ml of sodium chloride (0.15 m) respectively. This mixture was left to sit at 37°C for 25 minutes. Then, the test tubes containing the resultant supernatant, abundant in WBC, were centrifuged for 10 minutes at 5000 rpm. This process was performed twice. A neutrophil population of 85-87% was obtained using the differential WBC from the resultant WBC-rich cell pellet. 

Chemotaxis assay assembly

This assembly consists of two compartments, upper and lower. The former consists of a syringe (5 mm pore size) with calcium acetate filter paper at one end that contains the suspension of cells, while the latter consists of Hank’s balanced salt solution containing a chemoattractant. The upper compartment was placed inside the lower compartment. This assembly was made to rest at room temperature for about one hour (undisturbed). Post this, the upper compartment was emptied of its cell contents, and this was submerged in methanol (70%) allowing the glue to melt. The filter paper strip was detached with caution and stained in hematoxylin. This was then fixed on a glass slide and observed under a microscope. The statistical analysis was done using the IBM SPSS Statistics for Windows, Version 20.0 (Released 2011; IBM Corp., Armonk, New York, United States). The p-value was set at <0.001. This indicated statistical significance. The mean, as well as the standard deviation, were obtained for all the groups. Analysis of variance (ANOVA) test was used to aid in testing for differences in means amongst the categorical groups.

## Results

Comparison of neutrophil count (%) using a one-way ANOVA test showed that the mean value was highest in Group IV (72.535) followed by Group III (71.29), Group II (62.13), and least in Group I (58.15). This difference showed a test value of 14.917 and p-value of <0.001 and hence is significant statistically. (Table 1)

**Table 1:** Percentage of various neutrophil count (%) scores between the groups

Dependent variable	Groups	N	Mean	Standard deviation	p-value
Neutrophil count (%)	Group I	20	58.15	8.67	<0.001
	Group II	20	62.13	8.92	
	Group III	20	71.29	8.14	
	Group IV	20	72.53	6.47	
	Total	80	66.02	10.03	

On intergroup comparison there was a significant difference statistically noted between all the groups except between Group I and Group II, and between Group III and Group IV. (Table 2)

**Table 2:** Percentage of various neutrophil count (%) scores between the groups

Dependent variable	Comparison group	Compared with	Mean difference	Standard error	p-value
Neutrophil count (%)	Group I	Group II	-3.98	2.56	0.413
		Group III	-13.14	2.56	<0.001
		Group IV	-14.38	2.56	<0.001
	Group II	Group III	-9.16	2.56	0.003
		Group IV	-10.40	2.56	0.001
	Group III	Group IV	-1.24	2.56	0.962

Analogy of neutrophil chemotaxis using a one-way ANOVA test showed that the mean value of Group I (156.85) was highest, followed by Group II (149.55), Group III (118.45), and least in Group IV (81.3). This difference resulted in a test value of 39.685 and a p-value of <0.001, thus indicating that it is significant statistically (Table 3).

**Table 3:** Percentage of various neutrophil chemotaxis scores between the groups

Dependent variable	Groups	N	Mean	Standard. deviation	p-value
Neutrophil chemotaxis	Group I	20	156.85	10.35	<0.001
	Group II	20	149.55	6.63	
	Group III	20	118.45	14.84	
	Group IV	20	81.3	16.61	
	Total	80	126.53	32.51	

On intergroup analysis, a statistically significant difference was seen between all the groups except for Group I and Group II (Table 4).

**Table 4:** Percentage of various neutrophil chemotaxis scores between the groups

Dependent variable	Comparison group	Compared with	Mean difference	Standard error	p-value
Neutrophil chemotaxis	Group I	Group II	7.3	4.02	0.275
		Group III	38.40	4.02	<0.001
		Group IV	75.55	4.02	<0.001
	Group II	Group III	31.10	4.02	<0.001
		Group IV	68.25	4.02	<0 001
	Group III	Group IV	37.15	4.02	<0.001

The correlation between the blood parameters (neutrophil count and neutrophil chemotaxis) and the clinical parameters (plaque score, gingival score, calculus score, periodontal pocket depth, and loss of attachment clinically) showed no significant difference statistically, in all the groups. The correlation between the neutrophil count (%) and calculus score showed a negative correlation and was significant, the p-value being 0.042 (Tables 5, 6).

**Table 5:** Correlation of the blood parameters and the periodontal parameters in Group I

Group I	Parameters being correlated	N	Correlation(r)	p-value
	Neutrophil count (%) and plaque index	20	-0.436	0.055
	Neutrophil count (%) and calculus score	20	-0.172	0.468
	Neutrophil count (%) and periodontal pocket depth	20	0.025	0.915
	Plaque index and neutrophil chemotaxis	20	0.334	0.151
	Calculus score and neutrophil chemotaxis	20	0.079	0.742
	Periodontal pocket depth and neutrophil chemotaxis	20	0.218	0.357

**Table 6:** Correlation of the blood parameters and the periodontal parameters in groups II, III, and IV

Parameters being correlated	N	Correlation(r)	Percentage
Neutrophil count (%) and plaque index (Group II)	20	-0.267	0.255
Neutrophil count (%) and calculus score (Group II)	20	-0.458	0.042
Neutrophil count (%) and gingival score (Group II)	20	0.098	0.681
Neutrophil count (%) and periodontal pocket depth (Group II)	20	0.093	0.698
Plaque index and neutrophil chemotaxis (Group II)	20	-0.068	0.774
Calculus score and neutrophil chemotaxis (Group II)	20	-0.234	0.321
Gingival score and neutrophil chemotaxis (Group II)	20	0.24	0.308
Periodontal pocket depth and neutrophil chemotaxis (Group II)	20	0.14	0.557
Neutrophil count (%) and plaque index (Group III)	20	-0.416	0.068
Neutrophil count (%) and calculus score (Group III)	20	-0.318	0.172
Neutrophil count (%) and gingival score (Group III)	20	0.068	0.777
Neutrophil count (%) and periodontal pocket depth (Group III)	20	0.222	0.346
Neutrophil count (%) and clinical attachment loss (Group III)	20	-0.024	0.919
Calculus score and neutrophil chemotaxis (Group III)	20	0.186	0.433
Gingival score and neutrophil chemotaxis (Group III)	20	-0.092	0.699
Periodontal pocket depth and neutrophil chemotaxis (Group III)	20	0.209	0.376
Clinical attachment loss and neutrophil chemotaxis (Group III)	20	-0.058	0.808
Neutrophil count (%) and plaque index (Group IV)	20	0.429	0.059
Neutrophil count (%) and calculus score (Group IV)	20	-0.397	0.083
Neutrophil count (%) and gingival score (Group IV)	20	0.222	0.348
Neutrophil count (%) and periodontal pocket depth (Group IV)	20	0.146	0.538
Neutrophil count (%) and clinical attachment loss (Group IV)	20	0.269	0.252
Plaque index and neutrophil chemotaxis (Group IV)	20	0.043	0.856
Calculus score and neutrophil chemotaxis (Group IV)	20	0.011	0.965
Gingival score and neutrophil chemotaxis (Group IV)	20	0.064	0.79
Periodontal pocket depth and neutrophil chemotaxis (Group IV)	20	-0.271	0.248
Clinical attachment loss and neutrophil chemotaxis (Group IV)	20	0.298	0.201

## Discussion

Gingivitis and various forms of periodontitis are chronic inflammatory diseases mainly due to bacterial infection and altered host response. These are the two most prevalent diseases of the oral cavity in humans. The occurrence of periodontal diseases differs among various populations across the world, and it shows a collateral increase with an increase in age. Systemic conditions such as cardiovascular diseases, atherosclerosis, anemia, diabetes mellitus, and pre-term low birth weight in infants show a higher risk in patients with chronic periodontitis, as shown in various epidemiologic studies [8]. Some studies have shown that systemic hematologic changes can be observed in periodontal infections. Periodontitis results in the formation of cytokines, most distinctively, tumor necrosis factor-alpha (TNF-α), interleukin (IL)-1, and IL-6/ [9]. Such inflammatory cytokines cause anemia by reducing the production of erythropoietin [10].

The various risk factors for cardiovascular diseases include genetic, biomedical, behavioral, and lifestyle characteristics. Those firmly established include low-density lipoprotein (LDL), blood pressure, smoking, dietary habits, and physical inactivity. Likewise, the most prevalent disease of the oral cavity is periodontal infection. It can affect up to 90% of the world's population. Bacterial biofilm, or dental plaque, is the root cause of gingivitis, which is the milder form of periodontal infection. The correlation between periodontal disease and coronary heart disease could be due to a fundamental response characteristic, that results in an individual developing an increased risk for both periodontal disease and arteriosclerotic vascular disease (ASVD) [11]. Periodontopathic bacteria like *Porphyromonas gingivalis* and their by-products initiate inflammatory mechanisms that prove to be the main link between periodontal disease and ASVD. The propagation of atherosclerotic lesions, the promotion of infiltration of activated WBC, and the upregulation of endothelial cell activation are brought about by the adaptive immune response to the presence of these bacteria (*Porphyromonas gingivalis*) that, in turn, aggravates the expression of pro-inflammatory cells [12].

In the present study, 80 participants were included (40 males and 40 females) and all four groups contained 20 participants as per the inclusion and exclusion criteria. All four groups were demographically similar concerning age and sex distribution at the time of sampling. The measurement of clinical parameters and their comparison with each other and each blood parameter in each group was done. The neutrophil count is an independent predictor of coronary heart disease. This has been indicated in various epidemiological studies. The majority of the studies show a dose-response effect, as higher levels of WBC counts are correlated with a gradual increase in susceptibility to cardiovascular diseases [13,14]. A meta-analysis by Wheeler et al. states that ischemic heart disease is predicted more profoundly by neutrophil counts than other constituents [15].

In our study, neutrophil count (%) showed the highest mean value in the LAP group (72.535), followed by the chronic periodontitis group (71.29), the gingivitis group (62.13), and the least in the healthy group (58.15). The results obtained revealed a test value of 14.917 and a p-value <0.001, which is significant statistically. The correlation between neutrophil count (%) and the clinical parameters like various indexes including plaque, gingival and calculus, periodontal pocket depth, and loss of clinical attachment showed no significant difference statistically in all the groups. The correlation between the parameters of neutrophil count (%) and calculus score showed a good negative correlation and was significant with a p-value of 0.042 in the gingivitis group. The outcome of our study was similar to that of the study done by Frederiksson et al., where remarkably higher WBC counts were reported in non-smoking patients with periodontitis in comparison to healthy individuals who did not smoke (control group) [16]. Also, a study performed by Wakai et al. shows that an independent association between periodontal disease severity and WBC count is present [9].

Neutrophils play a crucial role in both defending and spreading periodontal diseases. This has been ascertained by various research projects in the recent past. Despite contradictory reports of faulty neutrophil functions, most of the reports state that neutrophil functions like adhesion, phagocytosis, intracellular killing, and chemotaxis are diminished most often in LAP and periodontitis as well. Several research articles have shown that neutrophils in LAP remain in a state of hyperactivity. The common etiology of this hyperactivity may be due to the genotype of a person, circulating, or environmental factors. It is these hyper-responsive neutrophils that experts deem to be an important reason for the destruction of periodontal tissues that is commonly observed in LAP. The contradictory reports regarding the role of neutrophils in periodontitis could be due to the differences in genetic diversity among the various populations across the globe [17].

In our study, neutrophil chemotaxis showed the highest mean value in Group I (156.85), followed by Group II (149.55), Group III (118.45), and the least in Group IV (81.3). This difference, with a test value, of 39.685 and a p-value <0.001, was statistically significant. The correlation between neutrophil chemotaxis and clinical parameters like plaque index, gingival index, calculus index, periodontal pocket depth, and loss of attachment clinically showed no significant difference statistically in all the groups. Thus, it was concluded that reduced neutrophil chemotaxis was observed in localized aggressive periodontitis patients, followed by patients with chronic periodontitis, compared to the gingivitis group and the healthy group. The outcome of the present study is similar to that of the study done by Bhansali et al., which concluded that neutrophils in individuals exhibiting LAP showed lower levels of chemotaxis in comparison to healthier individuals [17].

An increase in neutrophil count and neutrophil chemotaxis, as seen in this study, could indicate that these parameters can be taken as reliable biomarkers for the detection of periodontal disease and cardiovascular diseases. Therefore, our study reveals that it is possible to speculate that periodontal disease may result in the patients being susceptible to a risk of cardiovascular disease, albeit within the limitations of the current study, i.e., a relatively small sample size.

## Conclusions

Traditional periodontal diagnostic techniques provide finite knowledge regarding the periodontal health of a patient as well as the probability of periodontal disintegration. However, the present study about neutrophil count and chemotaxis presents satisfactory data on the current progression of the disease and can anticipate the likelihood of subsequent atherosclerosis and other cardiovascular diseases in periodontitis patients. The correlation with these inflammatory markers in periodontal disease might be a possible causal pathway in linking the connection between periodontitis and risk for cardiovascular diseases in these patients. One of the drawbacks of the study was that non-surgical therapy in periodontitis patients was not integrated. Another limitation was the relatively small sample size. Hence, further research to study the cellular and molecular mechanisms of these markers to elicit the risk for various cardiovascular diseases is needed to establish and confirm a definite link.
